# Prevention and Treatment of Influenza with Hyperimmune Bovine Colostrum Antibody

**DOI:** 10.1371/journal.pone.0013622

**Published:** 2010-10-26

**Authors:** Wy Ching Ng, Victor Wong, Brian Muller, Grant Rawlin, Lorena E. Brown

**Affiliations:** 1 Department of Microbiology and Immunology, The University of Melbourne, Parkville, Victoria, Australia; 2 Immuron Limited, North Melbourne, Victoria, Australia; University of Rochester School of Medicine, United States of America

## Abstract

**Background:**

Despite the availability of specific vaccines and antiviral drugs, influenza continues to impose a heavy toll on human health worldwide. Passive transfer of specific antibody (Ab) may provide a useful means of preventing or treating disease in unvaccinated individuals or those failing to adequately seroconvert, especially now that resistance to antiviral drugs is on the rise. However, preparation of appropriate Ab in large scale, quickly and on a yearly basis is viewed as a significant logistical hurdle for this approach to control seasonal influenza.

**Methodology/Principal Findings:**

In this study, bovine colostrum, which contains approximately 500 g of IgG per milking per animal, has been investigated as a source of polyclonal antibody for delivery to the respiratory tract. IgG and F(ab')2 were purified from the hyperimmune colostrum of cows vaccinated with influenza A/Puerto Rico/8/34 (PR8) vaccine and were shown to have high hemagglutination-inhibitory and virus-neutralizing titers. In BALB/c mice, a single administration of either IgG or F(ab')2 could prevent the establishment of infection with a sublethal dose of PR8 virus when given as early as 7 days prior to exposure to virus. Pre-treated mice also survived an otherwise lethal dose of virus, the IgG- but not the F(ab')2-treated mice showing no weight loss. Successful reduction of established infection with this highly virulent virus was also observed with a single treatment 24 hr after virus exposure.

**Conclusions/Significance:**

These data suggest that a novel and commercially-scalable technique for preparing Ab from hyperimmune bovine colostrum could allow production of a valuable substitute for antiviral drugs to control influenza with the advantage of eliminating the need for daily administration.

## Introduction

Influenza is a highly contagious acute respiratory disease. Seasonal epidemics can affect 5–15% of the population, leading to an estimated 3–5 million cases of severe illness and an average of 250,000–500,000 deaths annually (www.who.int). Infection is mainly confined to the upper respiratory tract and large airways and only on rare occasions is primary viral pneumonia observed. The infection usually lasts for about 7–10 days and is characterized by the sudden onset of high fever, myalgia, headache and severe malaise, non-productive cough, sore throat, and rhinitis. Most people recover within one to two weeks without requiring any medical treatment but the economic impact of related factors such as time off work is significant. In the elderly, young children or those with certain underlying medical conditions, severe complications such as pneumonia due to secondary bacterial infection can accompany influenza and pose a serious threat. Typically, influenza virus is transmitted from infected individuals through aerosols produced by coughing and sneezing or through contact with contaminated surfaces. Symptoms can appear as soon as a day after exposure.

Prevention of influenza virus infection through vaccination poses a significant challenge. The high mutation rate that occurs during replication of the virus and selection of neutralisation-escape variants by pre-existing antibodies, leads to the virus undergoing “antigenic drift” within populations, such that a particular influenza vaccine usually confers protection for only a few years. For this reason, the strain composition of the vaccine needs to be updated regularly, often with virus isolates circulating in the previous winter in the opposite hemisphere. The vaccine is formulated each season with two influenza type A strains and a type B strain that are predicted to be antigenically well matched with influenza virus strains that are expected for the coming influenza season. However, the need to vaccinate yearly is one contributing factor to poor uptake rates and, even if vaccinated, it is possible to fall ill; the vaccine is only about 70% efficacious in young healthy adults [1] and on occasion the chosen vaccine strain does not match the emerging virus making it significantly less effective [2]. Vaccination against influenza does remain an important preventative health measure for the elderly where it can provide a 60% reduction in morbidity and 70–80% reduction in influenza-related mortality (www.who.int).

For treatment and prophylaxis of influenza, two classes of antiviral drugs are available. The adamantanes [3], amantadine and rimantadine, are inhibitors of the M2 ion channel and interfere with viral uncoating inside the cell. Though relatively inexpensive, their use has been associated with toxicity and the rapid emergence of drug-resistant variants [4], which are already prevalent worldwide amongst seasonal strains [5], [6] as well as the recently emerged swine origin pandemic virus [7]. Inhibitors of the viral neuraminidase, oseltamivir and zanamivir [8] stop the efficient release of progeny virus from infected host cells, thus reducing cell-to-cell spread. Neuraminidase inhibitors initially appeared less likely to promote drug-resistance but oseltamivir-resistant virus has arisen after treatment of patients [9], [10] and are now widespread in circulating strains [11], [12] To be effective, the time of commencement of treatment with anti-influenza drugs must be within two days of illness onset and the treatment is ongoing throughout the infection [13].

Despite these measures, annual epidemics continue to cause substantial morbidity and mortality worldwide and additional control strategies to compliment existing methods would be of significant benefit. What we currently lack is a method of prevention or treatment that provides no risk of selection of resistant strains and does not rely on continuous administration. Passive immunotherapy may provide a suitable option to fill this niche. Patients with diseases as diverse as rabies, hepatitis and respiratory syncytial virus (RSV) have been successfully treated with antibody (Ab) derived from hyperimmune sera from human or other animal origin [14]. Passive Ab therapy has also been shown to provide prophylaxis for high-risk individuals. In animal models of influenza, anti-viral polyclonal antisera from a variety of species have been successful in treating and preventing infection [15], [16] showing proof-of-principle for this disease.

Monoclonal antibodies (MAbs) targeting the viral hemagglutinin (HA) have been actively pursued as a continuous source of highly specific neutralising Ab [17], [18]. However, individual MAbs only target a single epitope on the virus so cocktails of MAbs with different specificities need to be used in order not to drive drift of the virus and this becomes very costly. Redeveloping such reagents every few years would be prohibitive. A technique to mass-produce polyclonal Abs at a low cost has been developed by Immuron Ltd. The polyclonal Abs are from vaccinated cattle and derived from the colostrum, the first milk given by the cow after birth. Using this dairy harvesting system, large amounts of Ab-rich product can be economically derived with yields of about 500 g of polyclonal IgG per vaccinated animal. We report here on the prophylactic and therapeutic efficacy of hyperimmune colostrum-derived antibodies against influenza virus in a mouse model.

## Materials and Methods

### Ethics statement

All experimental procedures using mice were carried out using protocols approved by the Animal Ethics Committee of the University of Melbourne (project 0703485.1). Vaccination of cows was done with approval of the Immuron Animal Ethics Committee, convened and registered under Victorian law (projects A04 and A09).

### Preparation of colostrum samples

First milking colostrum was collected from cows following immunization with three doses, 2 weeks apart, of purified detergent-disrupted A/Puerto Rico/8/34 (PR8) influenza virus in a water-in-oil emulsion adjuvant (Seppic, Fairfield NJ, USA). First milking colostrum was also collected from non-immunized cows for use as a control. Colostrum samples were centrifuged at 10,000× g for 30 mins at 4°C to remove fat and cells. Defatted colostrum was pasteurized 63.5°C for 30 mins and then recentrifuged. Colostrum whey was prepared by adjusting the pH to 4.6 with an equal volume of 0.2M sodium acetate solution (pH 4.0) while mixing at 37°C. The liquid was then left at 37°C for 2 hr, cooled and centrifuged at 10,000× g for 30 mins to remove casein.

### Purification of F(ab')_2_ fragments

Part of the colostral whey was adjusted to pH 4.0 and incubated with pepsin for 20 hrs at 37°C using a modification of the method by Jones and Landon [19]. Increasing the pH to 7.0 stopped pepsin digestion. The digested material was then centrifuged at 10,000× g (4–12°C) to remove the precipitate. Tangential flow diafiltration was then performed using a 30,000 MW cut-off membrane to remove low molecular weight products and washed with diafiltration buffer (20 mM sodium phosphate, 150 mM NaCl, pH 6.0). The filtrated product was then passed through a Q-Sepharose column to remove pepsin and residual acidic aggregates. All unbound material which corresponds to F(ab')_2_ fragments was collected and stored at 4°C. The final product was diluted in PBS. Protein concentration was determined by measurement of absorbance at 280 nm with a spectrophotometer.

### Purification of IgG

Another part of the colostral whey was adjusted to pH 6.6 and then diafiltrated against phosphate buffered saline (PBS: 20 mM sodium phosphate, 150 mM NaCl pH 7.5). This whey was filtered to 0.45 µm prior to purification using Protein-G Sepharose chromatography, using PBS as running buffer and 50 mM citrate pH 2.6 as elution buffer. After elution, peak protein was neutralized with 1M Tris pH 8.0 and then diafiltrated against PBS.

### Polyacrylamide gel electrophoresis (PAGE)

One microgram of protein in sodium dodecyl sulfate (SDS) sample buffer was subjected to electrophoresis under reducing and non-reducing conditions in a 4–20% Tris-HEPES SDS-PAGE gel. Protein bands were detected with Coomassie blue G-250 staining.

### Hemagglutination inhibition (HI) assay

HI assays were performed by standard procedures [20] in round-bottom polystyrene microtitre plates (Nunc, Denmark) using 1% chicken red blood cells. Titers are expressed as the reciprocal of the highest dilution of sample that inhibited 4 hemagglutinating units (HAU) of virus.

### Virus neutralization (VN) assay

The assay utilises virus plaque formation in Madin-Darby canine kidney (MDCK) cells [21]. Monolayers of MDCK cells were prepared by seeding 1.2×10^6^ cells in 3 ml of RPMI medium containing 10% fetal calf serum in each well of the tissue culture 6-well plate (TPP, CSL Ltd) and incubating overnight at 37°C in 5% CO_2_. The monolayers were then washed with medium containing antibiotics before adding 135 µl of virus-Ab mixtures to each well, in duplicate. The virus-Ab mixtures comprised half-log serial dilutions of Ab in 200 µl mixed with an equal volume of influenza virus to provide a final concentration of about 200 plaque forming units/135 µl and incubated together for 30 min at 37°C. The mixtures were allowed to adsorb to the monolayers for 45 min, with shaking of the plates at 15 min intervals. Then 3 ml warmed (45°C) overlay medium consisting of Leibovitz L-15 with glutamine at pH 6.8 (Gibco Invitrogen Corporation, Victoria, Australia) supplemented with 4-(2-hydroxyethyl)-1-piperazineethanesulfonic acid buffer (0.01M at pH6.8), 0.028% (w/v) NaHCO_3_ (APS Finechem, NSW, Australia), 100 IU per ml penicillin (CSL Ltd), 100 µg per ml streptomycin (CSL Ltd), 0.1% (w/v) TPCK-treated trypsin (Worthington Biochemical Corp., Lakewood, NJ, USA) and 0.9% (w/v) agarose (Sigma Chemicals Co.) were added to each well. The plates were then incubated at 37°C in 5% CO_2_ for 3 days. Plaques on the monolayers were then counted without staining. The VN titer was expressed as the reciprocal of the highest dilution that reduced the number of plaques to 50% of the control value in the absence of Ab.

### Mice and influenza virus infection

Inbred 6- to 10- week BALB/c mice (H-2d) were bred and housed at the Animal Facility of the Department of Microbiology and Immunology at the University of Melbourne. Mice were infected with 50 pfu (sublethal dose) or 500 pfu (lethal dose) of influenza A/Puerto Rico/8/34 (PR8, H1N1) by the intranasal (i.n.) route, either to the upper or total respiratory tract. In the upper respiratory tract (URT) model, inoculum is given to the nares of unanaesthetized mice in a small volume (10 µl), which confines the inoculum to the nasal turbinates. For the total respiratory tract (TRT) model, 50 µl of inoculum is given to the nares of the mice under penthrane anesthesia.

### Activity of Ab *in vivo*

In the prophylaxis experiments, anesthetized mice were given 50 µl of the Ab preparations or PBS intranasally (i.n.) 1, 2, 3 or 7 days before PR8 virus infection. For the localized treatment of the upper respiratory tract, unanaesthetized mice were given 10 µl Ab preparations i.n. on day 1 or on days 1 and 3 post URT infection. For treatment of the entire respiratory tract, anesthetized mice were given 50 µl Ab on days 1 or 2 post TRT infection. Mice were killed by cervical dislocation, one day (for prophylaxis experiments) or 5 days (for treatment experiments) after infection with a sublethal dose of virus. Nasal turbinates (in URT infected mice) or lungs (in TRT infected mice) were removed and homogenized and infectious viral titres were determined by plaque assay. For the survival experiments, mice infected with a lethal dose of virus were culled if they had lost >20% of their starting body weight and additionally displayed clinical signs of illness such as inactivity or hunched appearance.

### Detection of bovine IgG in tissue homogenates and sera

Anesthetized mice were given 50 µl of the anti-PR8 Ab preparation intranasally. After various intervals (1, 12, 24 and 36 hr), mice were killed and nasal turbinates, lungs and sera were collected. The tissues were homogenised in 1 ml of RPMI and both supernatants and sera were tested by ELISA for the presence of bovine Ab using a Bovine IgG ELISA quantitation set from Bethyl Laboratories, Montgomery, Texas, USA.

### Statistical analyses

These were carried out using the software package Prism (v.4.0c) from GraphPad Software Inc. A one-way ANOVA test with 95% confidence interval was employed. P values were obtained by using a Tukey post-test.

## Results

### Characterization of colostrum-derived IgG and F(ab')_2_ preparations

**Figure 1:**
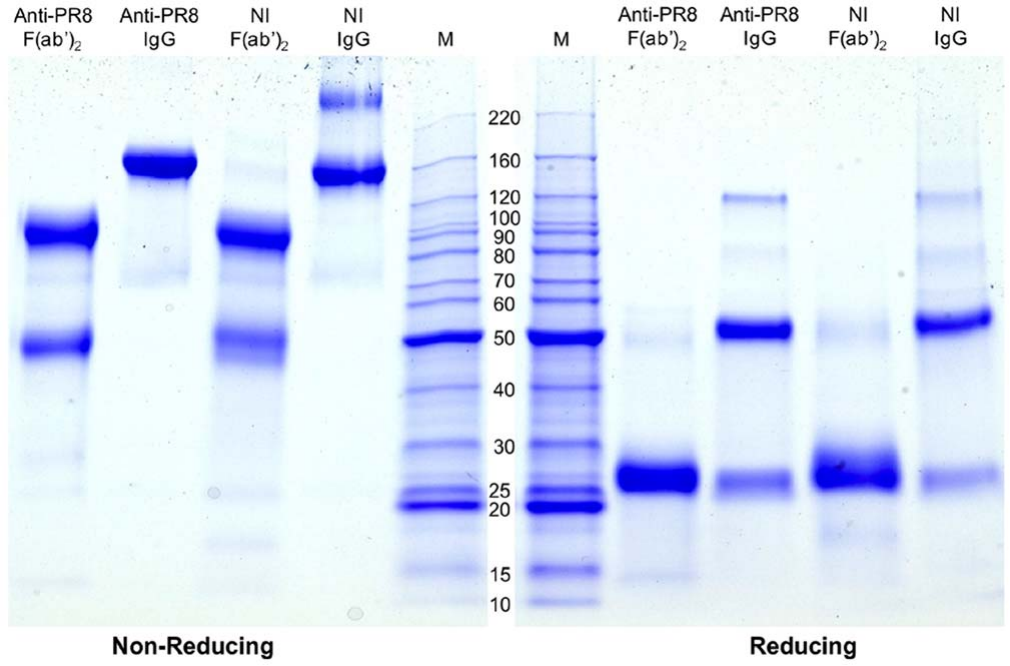
SDS-PAGE of IgG and F(ab')_2_ preparations. Protein samples in SDS buffer were subjected to electrophoresis under non-reducing and reducing conditions through a 4–20% Tris-HEPES SDS-PAGE gel. The gel was stained with Coomassie blue G-250. Molecular mass markers (M) are indicated in kilodaltons. NI, non-immune. The minor band in the non-immune IgG preparation run under non-reducing conditions corresponds to IgG dimers which can sometimes form after freeze-drying. The results are typical of profiles from three separate SDS-PAGE analyses of these particular preparations and of many other analyses of colostrum-derived antibodies .

The IgG and F(ab')_2_ preparations, derived from PR8-immune and non-immune colostrum samples, were subjected to SDS-PAGE (Fig. 1). Under non-reducing conditions, the IgG preparations migrated as a single major protein band with molecular mass of 160 kDa, while the F(ab')_2_ preparations migrated as a major band of 110 kDa, corresponding to F(ab')_2_ and a minor band at 55 kDa corresponding to F(ab'), present due to over-digestion with pepsin. Under reducing conditions, the IgG preparations migrated as two protein bands corresponding to the heavy chain (MW of 50 kDa) and light chain (25 kDa) while F(ab')_2_ migrated as a single band at 25 kDa.

The activity of the purified IgG and F(ab')_2_ preparations was tested in hemagglutination inhibition (HI) and virus neutralization (VN) assays against PR8 virus *in vitro*. Table 1 shows that the immune preparations both had high HI and NV titers whereas the non-immune control preparations showed no detectable activity in either assay.

**Table 1:** Hemagglutination inhibition and virus neutralization titers of Ab preparations.

Antibody preparation^a^	HI titer	VN titer
Anti-PR8 IgG	1280^b^	80,000
Anti-PR8 F(ab')_2_	2560	400,000
Non-immune IgG	<10	<10
Non-immune F(ab')_2_	<10	<10

a Antibody preparations were tested from a starting concentration of 20 mg IgG or F(ab')_2_ per ml.

b Mean of four determinations, each giving identical results.

### Therapeutic efficacy of anti-PR8 IgG and F(ab')_2_ in the URT

**Figure 2:**
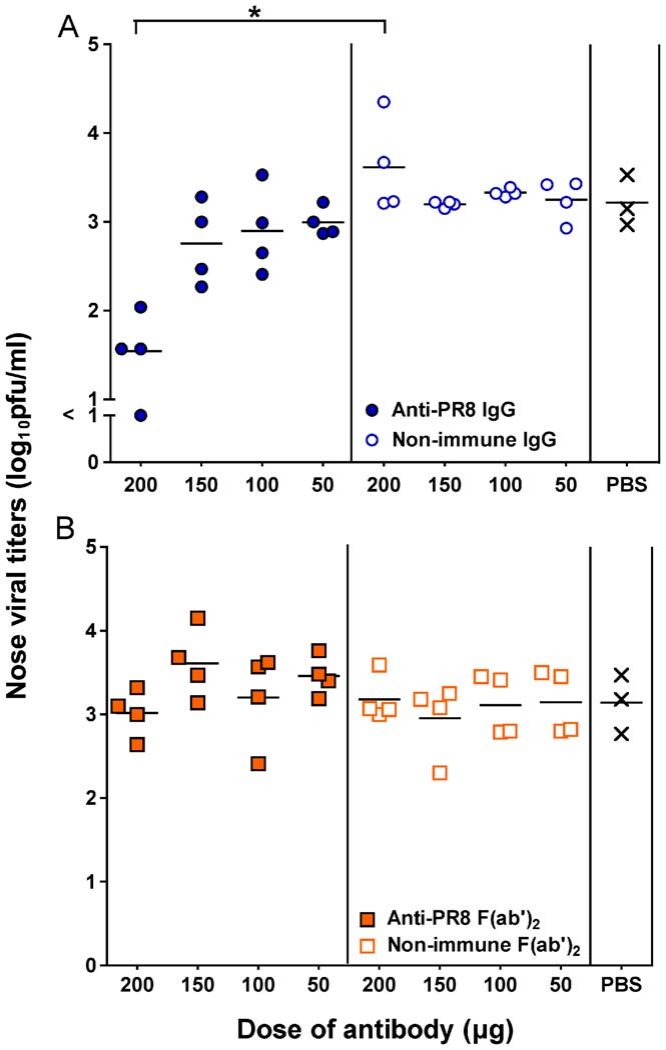
Virus loads are reduced in the nasal turbinates after treatment with the anti-PR8 IgG preparation. Unanesthetised BALB/c mice (n = 4) were given 500 pfu of PR8 virus in 10 µl by the intranasal route to establish a localised URT infection. Twenty four hours post-infection, mice were treated with 200 µg, 150 µg, 100 µg or 50 µg of (A) anti-PR8 IgG or non-immune IgG or (B) anti-PR8 F(ab')_2_ or non-immune F(ab')_2_ or PBS via the URT route. Five days post-infection, mice were killed and nasal turbinates were collected in 1 ml media. Viral titers were determined by plaque assay on MDCK cell monolayers. The experiment was performed once with 4 mice per group, the organs assayed in quadruplicate and titers averaged to provide a value for that animal. Each symbol represents an individual mouse and the line represents the mean of each group. The bracket with an asterisk indicates a statistically significant difference between the indicated groups.

The effect of the purified preparations on influenza virus *in vivo* was examined using a mouse model. Firstly, we performed a dose titration of the Ab preparations by treating mice with 50 µg, 100 µg, 150 µg or 200 µg of anti-PR8 IgG, anti-PR8 F(ab')_2_, non-immune control Abs or PBS via the URT route, 24 hrs after establishment of infection with PR8 virus. Nasal turbinate viral titres were determined 5 days post-infection (4 days post-treatment) when normally still at their peak in untreated animals. As shown in Figure 2A, 200 µg of the immune IgG preparation resulted in a 100-fold reduction in viral titers compared to the PBS-treated mice (p<0.001). Lower doses showed no statistically significant differences compared to the PBS group. The immune F(ab')_2_ preparation did not show any reduction in viral loads at any of the doses tested (Fig. 2B).

**Figure 3:**
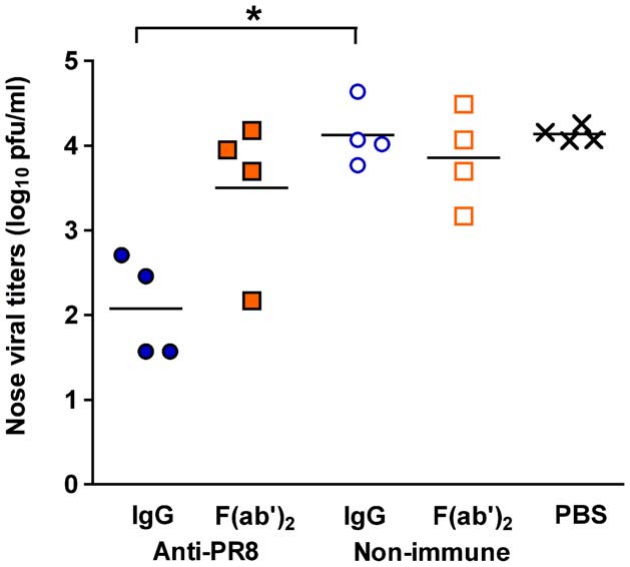
Virus load reduction is not enhanced by a second dosing with Ab. BALB/c mice were given 500 pfu of PR8 virus in 10 µl by the intranasal route to establish a localised URT infection. One and 3 days post-infection, mice were treated with 200 µg of each Ab preparation or PBS via the URT route. Five days post-infection, mice were killed and nasal turbinates were collected in 1 mL media. Viral titers were determined by plaque assay on MDCK cell monolayers. The experiment was performed once with 4 mice per group, the organs assayed in quadruplicate and titers averaged to provide a value for that animal. Each symbol represents an individual mouse and the line represents the mean of each group. Brackets with an asterisk indicate a statistically significant difference between the indicated groups.

A limitation of the URT route model is that only a small volume can be administered, prohibiting treatment with larger doses of Ab at any one time. Therefore, we investigated whether multiple administrations would further aid in the suppression of viral replication. On days 1 and 3 post PR8 virus infection, mice were treated with 200 µg of immune IgG, immune F(ab')_2_, or non-immune control preparations or PBS via the URT route. Figure 3 shows that the viral titres in the immune IgG-treated animals were significantly lower than their control counterparts 5 days post-infection. However, the dose response pattern was essentially no different from a single dose treatment regime. Furthermore, two doses of anti-PR8 F(ab')_2_ was still insufficient to reduce viral titres in the nasal turbinates.

### Therapeutic activity of anti-PR8 IgG and F(ab')_2_ in the TRT

**Figure 4:**
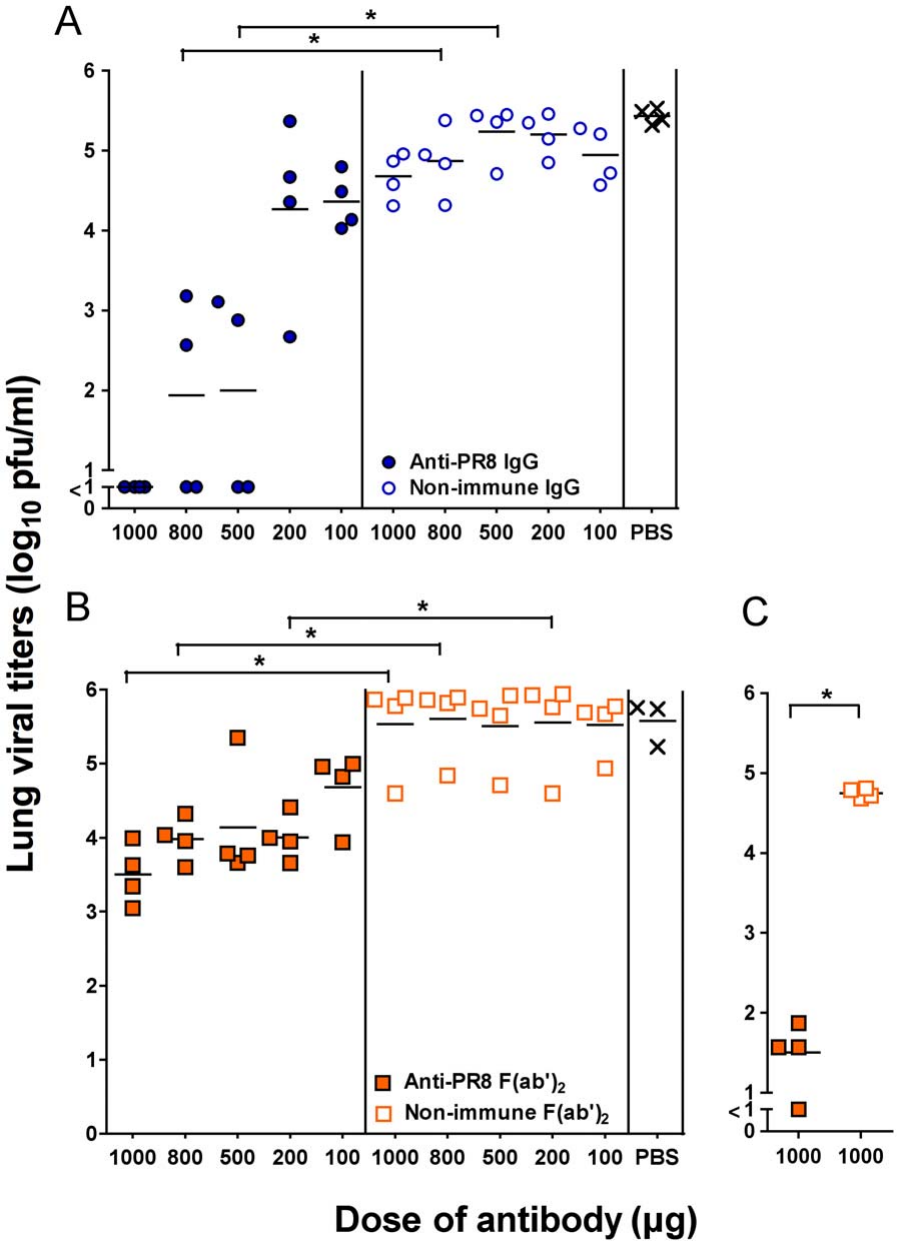
Virus loads are reduced in the lungs after treatment with the anti-PR8 IgG and F(ab')_2_ preparations. BALB/c mice (n = 4) were infected with 50 pfu of PR8 virus in 50 µl via the TRT route under penthrane anaesthesia. Twenty four hours post-infection, mice were treated with 1000 µg, 800 µg, 500 µg, 200 µg or 100 µg of (A) anti-PR8 IgG or non-immune IgG or (B) anti-PR8 F(ab')_2_ or non-immune F(ab')_2_ or PBS via TRT route. Five days post-infection, mice were killed and lungs were collected in 1 ml media. (C) Mice were infected and treated as in (B) and lungs sampled 7 days post infection. Viral titres were determined by plaque assay on MDCK cell monolayers. The experiment was carried out in its entirety once and results are consistent with repeat experiments on selected doses. Each symbol represents an individual mouse and the line represents the mean of each group. Brackets with an asterisk indicate a statistically significant difference (p<0.001) between the indicated groups.

We took a similar approach to investigating the ability of different concentrations of Ab to clear virus from the lungs of infected mice after TRT delivery. As with the URT model, the non-immune IgG and F(ab')_2_ preparations delivered by the TRT route 24 hrs after virus infection provided no reduction of viral loads in the lungs (Fig. 4A and B). However, 100% of mice treated with 1 mg of anti-PR8 IgG had undetectable viral titers in the lungs 5 days later (Fig. 4A). Half of the mice treated with 500 µg and 800 µg of anti-PR8 IgG completely cleared lung virus and the remainder showed partial clearance, with the mean viral titers of these groups significantly lower than the PBS control mice (p<0.001 for both). When mice were treated with lower concentrations of immune IgG (100 µg and 200 µg), no significant decreases in lung viral titres were observed. Treatment with equivalent amounts of anti-PR8 F(ab')_2_ did not provide complete clearance at any of the doses tested (Fig. 4B). Nevertheless, significant partial clearance was observed, particularly at the highest dose where 100-fold less virus was present in the lungs on day 5 (p<0.001), and also at 800, 500 and 200 µg doses (p<0.05 for all three).

Of interest was whether the decreased pulmonary viral loads seen 5 days after virus infection in mice treated with 1 mg of the immune F(ab')_2_ preparation might enable the mouse's own developing immune system to clear the virus more rapidly. Typically, this sublethal dose of PR8 will not be completely cleared in untreated animals until day 10 post infection. As shown in Figure 4C, by day 7 post-infection, lung viral titres in anti-PR8 F(ab')_2_-treated mice have decreased over 1000-fold compared to non-immune F(ab')_2_-treated mice, with 1 mouse showing undetectable viral titres. This implies an increase in the rate of overall viral clearance of 2–3 days after a single dose of immune F(ab')_2_.

**Figure 5:**
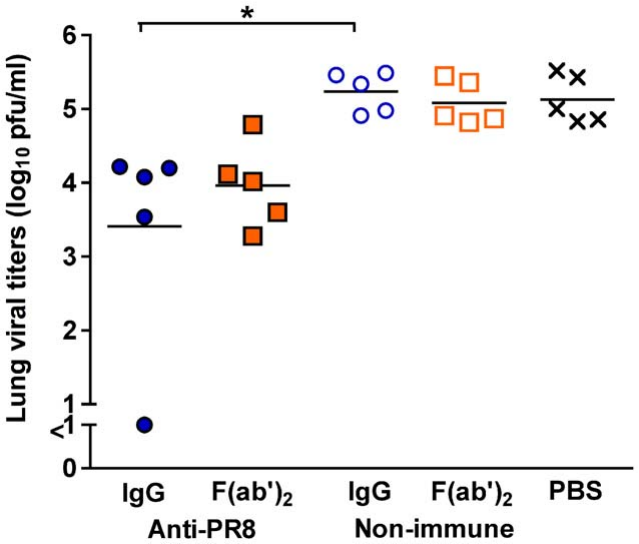
Virus loads are reduced but by a lesser extent if Ab treatment is delayed. BALB/c mice were infected with 50 pfu of PR8 virus in 50 µl via the TRT route under penthrane anaesthesia. Two days post-infection, mice were treated with 1 mg of the Ab preparations or PBS via the TRT route. Five days post-infection, mice were killed and lungs were collected in 1 ml media. Viral titres were determined by plaque assay on MDCK cell monolayers. The experiment was performed once with 5 mice per group, the organs assayed in quadruplicate and titers averaged to provide a value for that animal. Each symbol represents an individual mouse and the line represents the mean of each group. Brackets with an asterisk indicate a statistically significant difference between the indicated groups.

Treatment with the immune antibody preparations on day 2 post infection gave somewhat less of an effect (Fig. 5). Nevertheless, a reduction in viral titres was seen in the anti-PR8 IgG treated mice (mean 50-fold reduction, p<0.05) and also in the F(ab')_2_-treated mice (mean 10-fold reduction), although the latter was not statistically significant by the ANOVA test used.

### Prophylactic efficacy of anti-PR8 IgG and F(ab')_2_ in the total RT

**Figure 6:**
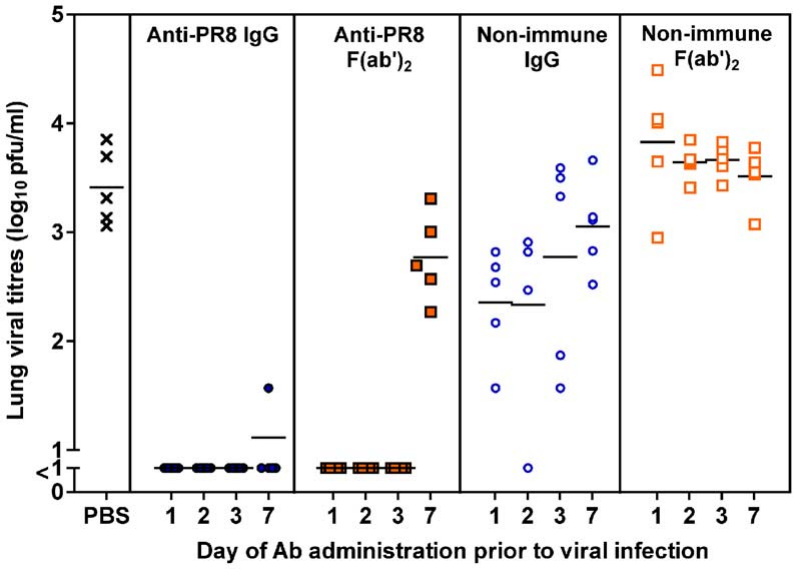
Anti-PR8 IgG and F(ab')2 preparations can completely prevent influenza infection. BALB/c mice (n = 5) were given 1 mg of the Ab preparations or PBS via the TRT route in 50 µl under penthrane anaesthesia. One, 2, 3 or 7 days later, mice were infected with 50 pfu of PR8 virus in 50 µl via the TRT route under penthrane anaesthesia. One day post-infection, mice were killed and lungs were collected in 1 ml media. Viral titres were determined by plaque assay on MDCK cell monolayers. Each symbol represents an individual mouse and the line represents the mean of each group. The experiment was carried out in its entirety once and results are consistent with repeat experiments on selected doses.

The activity of the Ab preparations was then investigated in a prophylactic setting (Fig. 6). Anti-PR8 IgG, anti-PR8 F(ab')_2_, non-immune Ab or PBS were given via the TRT route 1, 2, 3 or 7 days prior to infection with a non-lethal dose of PR8 virus. One day later the lungs of the mice were examined for infectious virus. Anti-PR8 IgG given as early as 3 days prior to infection completely inhibited virus growth in the lungs. This was also true for 4 of 5 mice given the immune IgG seven days prior to viral infection, with the remaining animal showing very low titers, less than 1/100^th^ of the PBS controls. Anti-PR8 F(ab')_2_ also completely prevented viral growth when given up to 3 days before infection. However, anti-PR8 F(ab')_2_ was not as efficient in preventing infection compared to anti-PR8 IgG when given 7 days prior to infection. Nonetheless, anti-PR8 F(ab')_2_-treated mice showed lower viral titres than PBS control mice.

Of particular interest was the observation that non-immune IgG (but not non-immune F(ab')_2_) was also able to lessen viral loads in the lungs, by approximately 10-fold if delivered one (P<0.05) or 2 (p<0.01) days prior to infection.

### Ability of colostrum derived Abs to counter lethal infection

**Figure 7:**
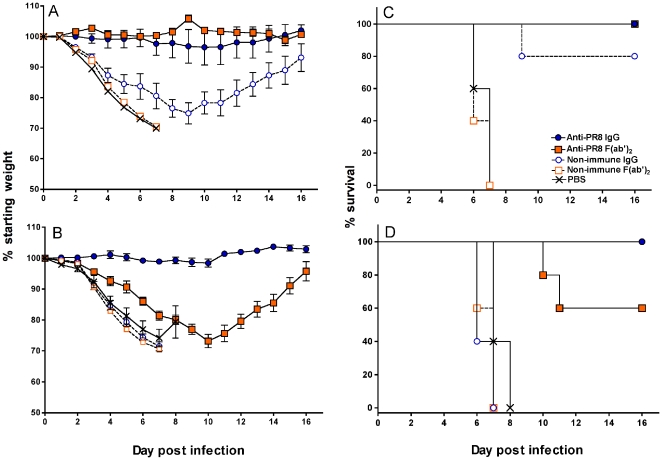
Anti-PR8 IgG and F(ab')_2_ preparations can prevent severe weight loss and death from a lethal dose of PR8 virus. BALB/c mice (n = 5) were treated with 1 mg of the Ab preparations or PBS in 50 µl i.n. under penthrane anaesthesia. On days 3 (A and C) or 7 (B and D) after Ab administration, mice were infected with 500 pfu of PR8 virus in 50 µl via the TRT route under penthrane anaesthesia. Mice were monitored daily for 16 days and killed at the humane endpoint. The experiment was performed once in accordance with animal ethics approvals. The change in percentage of the starting weight (A and B) and Kaplan-Meier survival curves (C and D) are shown.

**Figure 8:**
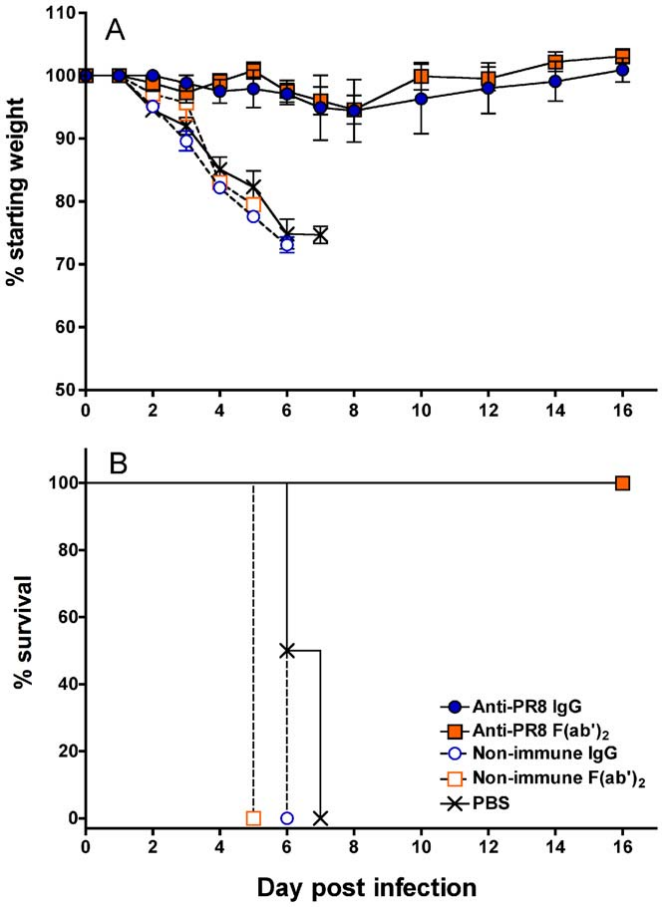
Anti-PR8 IgG and F(ab')_2_ preparations can prevent severe weight loss and death from an established lethal PR8 infection. BALB/c mice (n = 4) were infected with a lethal dose (500 pfu in 50 µl) of PR8 virus via the TRT under penthrane anaesthesia. One day post-infection, mice were treated with 1 mg of the Ab preparations or PBS in 50 µl via the TRT route. Mice were monitored daily for 16 days and killed at the humane endpoint. The experiment was performed once in accordance with animal ethics approvals. The change in percentage of the starting weight (A) and Kaplan-Meier survival curves (B) are shown.

After successful demonstration of the ability of the immune colostrum preparations to reduce or inhibit infection with a sub-lethal dose of influenza virus *in vivo*, we went on to assess their ability to counter infection with a lethal dose of PR8 virus. Anti-PR8 IgG, F(ab')_2_, the non-immune counterparts or PBS were given before (Fig. 7) or after (Fig. 8) PR8 infection and the mice monitored over a period of 16 days post infection for weight loss. Where necessary, mice were culled at the pre-determined humane endpoint. When the Ab preparations were given 3 days before infection (Fig. 7A and C), anti-PR8 IgG or F(ab')_2_-treated mice not only survived but maintained their bodyweights. Non-immune IgG-treated mice initially lost substantial weight but otherwise did not show signs of severe infection; all but one mouse (that was culled) eventually regained weight. In contrast, non-immune F(ab')_2_ and PBS treated mice rapidly lost weight, showed clinical signs of severe infection and had to be culled by day 7.

When the Ab preparations were administered 7 days before infection (Fig. 7B and D), all groups of mice, except the group treated with anti-PR8 IgG, lost weight. The average rate of weight loss in the anti-PR8 F(ab')_2_-treated mice was slower, however, and 3 of the 5 mice continued to be active and eventually regained weight; the other two were culled on days 10 and 11. Mice treated with non-immune preparations behaved similarly to PBS control mice, rapidly losing weight and becoming inactive prior to culling on days 6–8.

Treatment with a single dose of anti-PR8 IgG or F(ab')_2_ 24 hr after lethal infection (Fig. 8) completely prevented weight loss, severe disease and death. Mice that were given non-immune Ab or PBS rapidly lost weight over a period of 6 days, displayed severe clinical signs and had to be killed between days 5 and 7 post-infection.

### Bovine IgG is retained for long periods in the lungs of mice

**Figure 9:**
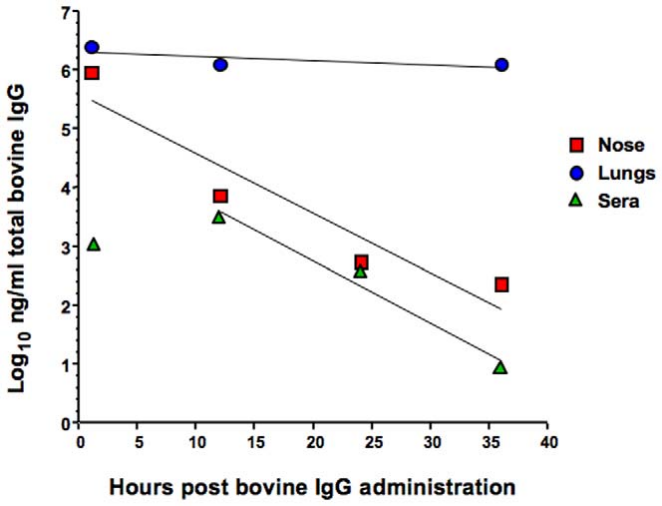
Half-lives of intranasally-delivered bovine IgG in the nose, lungs and serum. The bovine anti-PR8 IgG preparation (1 mg) was administered in a volume of 50 µl by the i.n. route to anesthetized mice. At 1, 12, 24 and 36 hr post-administration, mice were killed and bovine IgG was detected by capture ELISA in nasal turbinate and lung homogenates and in sera. The total amount of Ab was determined by reference to a standard curve. Linear regression analysis was used to calculate the half-lives of bovine IgG in the different sites. The data are representative of two experiments.

The ability of the anti-PR8 IgG preparation to prevent weight loss and death when given 7 days prior to lethal infection suggested that the Ab either remained within the RT for long periods or entered the bloodstream and was recruited back into the RT upon inflammation due to viral infection. To investigate the persistence of bovine Ab, mice were anesthetized and given 1 mg anti-PR8 IgG preparation in 50 µl i.n. After 1, 12, 24 and 36 hr, mice were killed and supernatants of homogenised nasal turbinates and lungs as well as sera were assayed in a capture ELISA for the presence of bovine IgG (Fig. 9). The results show that a very small amount of IgG does make its way into the bloodstream, peaking sometime between 1 to 12 hr but then is cleared with a half-life of about 15 hr. High levels in the nose are not maintained and are rapidly cleared with a half-life of about 4 hr. In contrast, bovine IgG persists in the lungs at high levels with a calculated half life of at least 36 hr.

## Discussion

For this study, highly active preparations of IgG and F(ab')_2_ containing polyclonal antibody with specificity for influenza virus PR8 were prepared from the colostrum of vaccinated cows. To our knowledge, few studies have demonstrated the effects of hyperimmune polyclonal antibody for prevention and treatment of influenza infection when given via the intranasal route [22]. When tested in mice with established PR8 infection of the nose, we showed that 200 µg of the IgG preparation could lead to significant reduction of infectious virus at that site. Similar effects in humans would imply a reduced rate of shedding and potential decrease in transmission, providing a community as well as a personal benefit. At the doses tested, however, the anti-PR8 F(ab')_2_ showed no such effect on URT infection despite this preparation having similar anti-viral titres *in vitro*. This may have been due to the additional Fc-dependent functions of IgG [23], [24], such as Ab-dependent cellular cytotoxicity or Ab plus complement lysis of infected cells, but may equally be a function of the slightly increased longevity of intact IgG (3× over Fab) in the respiratory tract [25]. Treatment with a second dose of Ab two days later did nothing to improve the reduction in viral loads mediated by IgG. This may suggest that once the virus loads are set early in infection by the first dose of antibody, the impact of a second dose of antibody later on is minimal compared to the viral clearance mediated by the developing immune response.

Treatment of the entire respiratory tract with 1 mg of anti-PR8 IgG given 24 hr after a sub-lethal infection resulted in total clearance of virus from the lungs measured on day 5 after infection. If treatment was delayed for a further 24 hrs, somewhat less impressive but nevertheless significant clearance levels (100-fold) were achieved. An effect on viral loads was also seen with the anti-PR8 F(ab')_2_ preparation, possibly because of the greater doses that could be tested via this route of delivery compared to URT delivery. The reduction in viral loads after treatment with F(ab')_2_ resulted in mice being able to clear the virus about 2–3 days earlier than untreated mice. In this respect, treatment with only a single dose of F(ab')_2_ gave similar benefits to continued administration of anti-viral drugs [13]. Importantly, treatment with 1 mg of either anti-PR8 IgG or F(ab')_2_ 24 hr after a lethal dose of PR8 not only prevented death of all animals but also prevented development of severe disease with minimal weight loss.

Testing of the antibody preparations for their prophylactic effects revealed that a single dose of anti-PR8 IgG, given up to 7 days before PR8 infection, was able to completely prevent the establishment of pulmonary infection in mice challenged with a sub-lethal dose of virus, and also completely protect against weight loss and death following a lethal dose of the virus. The long lasting effects of the bovine IgG is compatible with its half-life of >36 hr in the lungs, which contrasts with the shorter half-life observed in the nasal tissue and serum. Anti-PR8 F(ab')_2_ was also able to completely prevent virus establishment in the lungs and a lethal outcome but the longevity of this effect after a single dose was not as great as observed with IgG.

When translated into human use, it is likely that the duration of the prophylactic effect seen with IgG will be more comparable to what we have seen here with F(ab')_2_. The longevity of bovine IgG in mice, as seen with day 7 prophylaxes, may be attributed to the function of neonatal Fc receptors (FcRn) which prevent circulating IgG from degradation by transporting it within and across cells [26], although this effect is much less pronounced with IgG delivered intranasally [25]. Mouse FcRn has been shown to be highly promiscuous as it binds Fc of different species including bovine IgG, whereas human FcRn binds less well to IgG from this species [27]. For systemic application of therapeutic Abs the issue of longevity is important for reducing the number of doses required but is also a double-edged sword. The host will eventually develop an immune response against the Fc portion of an administered whole Ab of a different species, causing hypersensitivity reaction [28], [29]. Development of effective F(ab')_2_ for prophylaxis and treatment has therefore become increasingly important and delivery of this product by the intranasal route rather than systemically may provide additional safety.

In the prophylaxis experiment, a surprising observation was that non-immune IgG, but not non-immune F(ab')_2_, had a short term role in partially reducing pulmonary viral titres and death, though not morbidity. This is in contrast to the use of non-immune Ab for therapy of an established infection where it had no effect. Although the non-immune IgG showed no HI or VN activity *in vitro*, it may contain low levels of Ab that can act upon virions or infected cells by Fc-dependent mechanisms *in vivo*. This is supported by the observation of low levels of binding of the non-immune IgG to PR8 virus in ELISA (data not shown). The appearance of such Ab in the colostrum of unvaccinated cows may indicate cross-reactivity with other antigens encountered by this host.

The gathering of antibodies from bovine colostrum and their use in controlled clinical studies for a variety of infectious diseases, particularly for oral use against gastrointestinal diseases [30], has shown both prophylactic and therapeutic effects [31]. The production systems are well known in areas of the dairy industry and colostrum is produced in tonne volumes for use as a food supplement in many markets through the world. The techniques of vaccinating dairy cattle in large numbers in strategic campaigns is also well known to the veterinary profession and farmers as cattle are routinely vaccinated against cattle pathogens. An additional course of killed adjuvanted vaccines targeting a human pathogen can easily be applied within modern animal husbandry routines and IgG can be collected with variations of routine dairy techniques. Colostrum from cattle is very high in IgG as it is the main route of transfer of the immune proteins to the calf, which can directly absorb these large proteins to the blood within the first 24 hours [31]. The modern dairy cow produces much more colostrum that a single calf requires.

Use of colostrum-derived Ab in our study not only provides large amounts of specific Ab (estimated as sufficient for at least 500 human doses from each cow) but the antibody is polyclonal and can easily be converted to IgG and F(ab')_2_. This study builds on previous observations on the use of F(ab')_2_ to successfully treat influenza infection [16], even in severe combined immunodeficiency (SCID) mice [18], [25]. The F(ab')_2_ used in those studies, however, was derived from a MAb preparation. With all Ab being of the desired specificity, only a relatively low dose was required to clear virus. The dose of Ab used in our study is necessarily large as much of the antibody is non-specific. However the polyclonal Ab approach has the added advantage of providing broader antigenic coverage. This should have less effect in driving selection of resistance and the preparations will be expected to have an increased lifespan for use against naturally occurring drift variants due to any neutralizing Ab against epitopes that persist for several years. Such epitopes cannot be predicted with certainty. Current strategies using MAbs aim to prepare multi-specific preparations by combining several MAbs as a ‘cocktail’ [32], [33], which may overcome these hurdles but increases the expense, which is already high for large-scale production. Human anti-influenza MAbs have been successfully prepared and tested in mice for use against H5N1 infection [33] but for mouse-derived MAbs, the additional step of “humanizing” mouse-derived MAbs to reduce hypersensitivity [32] for parenterally delivered preparations also increases cost and lead time to product availability. Furthermore, technologies that rely on delivering Ab preparations with only a small number of specificities selected prior to or at the beginning of an epidemic may lose protective capacity during the course of an epidemic in certain individuals as the virus evolves.

This study shows the potential of bovine colostrum-derived polyclonal antibodies or their fragments delivered to the respiratory tract for both treatment and prevention of influenza. Due to the relative low cost and high volume capability of production, this new approach represents a significant tool for individual and large-scale public health management of influenza in humans. The process, which takes roughly 4 months from first vaccination of naïve cows to final product has the potential to be significantly shortened if the same animals were re-boosted with the latest strain. This may also select for broadly crossreactive antibodies which would have a significant advantage if the vaccine was not a perfect match for the emerging virus, as we see occasionally with vaccines for human use.

The product would have significant advantages over the use of antiviral agents due to the lack of need for daily administration and may be a preferred control measure against drug-resistant viruses. In those who have suboptimal protection against influenza through not being previously vaccinated or those vaccinated individuals who fail to adequately seroconvert, especially the elderly or those with compromised immune systems, treatment with polyclonal Abs may have a significant role in reducing morbidity and mortality.
